# Comparative Analysis of Root Transcriptome Reveals Candidate Genes and Expression Divergence of Homoeologous Genes in Response to Water Stress in Wheat

**DOI:** 10.3390/plants9050596

**Published:** 2020-05-07

**Authors:** Behnam Derakhshani, Habtamu Ayalew, Kohei Mishina, Tsuyoshi Tanaka, Yoshihiro Kawahara, Hossein Jafary, Youko Oono

**Affiliations:** 1 Department of Agronomy & Plant Breeding, Faculty of Agriculture, University of Zanjan, Zanjan 45371-38791, Iran; Behnam.derakhshani@znu.ac.ir; 2 Breeding Material Development Unit, Institute of Crop Science, National Agriculture and Food Research Organization (NARO), Tsukuba 305-8518, Japan; 3 Small Grains Breeding Laboratory, Noble Research Institute LLC, Ardmore, OK 73401, USA; habtamu01@gmail.com; 4 Plant Genome Research Unit, Institute of Crop Science, NARO, Tsukuba 305-8518, Japan; mishina@affrc.go.jp; 5 Breeding Informatics Research Unit, Institute of Crop Science, NARO, Tsukuba 305-8518, Japan; tstanaka@affrc.go.jp (T.T.); y.kawahara@affrc.go.jp (Y.K.); 6 Bioinformatics Team, Advanced Analysis Center, NARO, Tsukuba 305-8518, Japan; 7 Iranian Research Institute of Plant Protection, Agricultural Research, Education and Extension Organization (AREEO), Tehran 19395-1454, Iran; h.jafary@areeo.ac.ir

**Keywords:** wheat, water stress, abiotic stress, homoeolog gene expression, RNA-Seq

## Abstract

Crop cultivars with larger root systems have an increased ability to absorb water and nutrients under conditions of water deficit. To unravel the molecular mechanism of water-stress tolerance in wheat, we performed RNA-seq analysis on the two genotypes, Colotana 296-52 (Colotana) and Tincurrin, contrasting the root growth under polyethylene-glycol-induced water-stress treatment. Out of a total of 35,047 differentially expressed genes, 3692 were specifically upregulated in drought-tolerant Colotana under water stress. Transcription factors, pyrroline-5-carboxylate reductase and late-embryogenesis-abundant proteins were among upregulated genes in Colotana. Variant calling between Colotana and Tincurrin detected 15,207 SNPs and Indels, which may affect protein function and mediate the contrasting root length phenotype. Finally, the expression patterns of five triads in response to water, high-salinity, heat, and cold stresses were analyzed using qRT-PCR to see if there were differences in homoeologous gene expression in response to those conditions. The five examined triads showed variation in the contribution of homoeologous genes to water, high-salinity, heat, and cold stresses in the two genotypes. The variation of homoeologous gene expression in response to environmental stresses may enable plants to better cope with stresses in their natural environments.

## 1. Introduction

Wheat was one of the first cereals to be domesticated, and it provides essential nutrition to more than one third of the population worldwide. The annual demand for cereals has been on the rise for many years now, necessitating a 60% increase in wheat production to meet global food and fiber needs by the year 2050 [1,2]. However, achieving this goal involves great challenges exacerbated by climate change, which causes significant water deficits and abnormally high temperatures, and increased drought severity as a result [3]. Drought is one of the most important environmental stresses leading to low yield and quality of wheat [4].

Phenotyping for drought-stress tolerance is notoriously variable depending on its time of occurrence during the crop cycle, severity and duration, and co-occurrence with other environmental stress factors such as high or low temperatures. Understanding the overall physiobiochemical coping mechanisms of plants and incorporating genetic markers into breeding planning will help to better design parental line selection [5,6,7].

Plants respond to water stress through various morphological and physiochemical adjustments. Roots are the first plant organs that perceive water-deficit signals, followed by initiation of a cascade of gene expression responses leading to stress tolerance [8,9]. Deep rooting is the widely accepted trait for drought resistance improvement, since it helps to extract water stored in deep soil profiles [10,11]. Studies have shown that deeper root systems improve water uptake and yield performance of various crops under drought-stress conditions [10,11,12]. Polyploid species often display more vigorous growth than their progenitor species [13]. In addition, species with higher states of ploidy often possess better abiotic-stress tolerance than their progenitors [14]. Mechanisms of increased abiotic-stress tolerance in polyploid species may involve polyploidy-contributed heterosis and expression dosage due to increased gene copy number [15]. However, the actual physiochemical changes that enable water-stress resistance remain a poorly understood area in wheat genetics and breeding. Water-limiting environments trigger various transcriptional, physiological, and developmental changes in plants. Profiling differential gene expression of genotypes at different levels of water availability gives insight regarding which genes are instrumental in response to water stress.

High-throughput transcriptome sequencing is a powerful tool for gene expression analysis. Recent studies detected water-stress-responsive genes in wheat using comparative transcriptome profiling [16,17,18,19]. The present study evaluated the transcriptome profiles of the two contrasting genotypes, Colotana 296-52 (Colotana) and Tincurrin, for root length under water stress. Colotana was among the genotypes with longer roots, while Tincurrin grew shorter roots under mild to severe water stress [20,21]. One study reported that abiotic stress treatments such as cold, dark, and water submersion altered homoeologous gene expression in allopolyploid cotton (*Gossypium hirsutum*) [22]. The objective of this research was to detect root-growth-responsive genes under water-stressed conditions that drive the phenotypic variation observed between the two genotypes, and to determine how homoeologous gene expression patterns change in response to abiotic stress conditions.

## 2. Results

### 2.1. RNA-Seq Analysis of Colotana and Tincurrin Roots under Water Stress

Relative changes in transcriptomic profiles of Colotana and Tincurrin genotypes were evaluated under a no-treatment (control) condition and a polyethylene glycol (PEG)-induced water-stress condition. The RNA-seq data from Colotana under the control condition (Colotana.con) and PEG-induced water-stress condition (Colotana.PEG), Tincurrin under control condition (Tincurrin.con) and PEG-induced water-stress condition (Tincurrin.PEG) generated a total of 432 million paired-end reads. Removal of low-quality reads resulted in total 398 million clean reads. An average of 84.93% of clean reads mapped to the wheat reference genome using the Tophat2 tool in both genotypes (Supplementary Table S1). Pearson’s correlation (Supplementary Figure S1A) and PCA plots (Supplementary Figure S1B) showed a high correlation among the replicates in each genotype.

**Figure 1:**
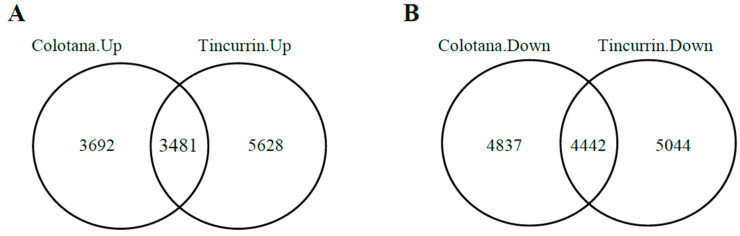
Venn diagram analysis of up- and downregulated genes (**A**) PEG-induced water-stress condition (Colotana.PEG) vs. Colotana under the control condition (Colotana.con) upregulated relative to PEG-induced water-stress condition (Tincurrin.PEG) vs. Tincurrin under control condition (Tincurrin.con) upregulated; (**B**) Colotana.PEG vs. Colotana.con downregulated relative to Tincurrin.PEG vs. Tincurrin.con downregulated.

To identify differentially expressed genes (DEGs) responsive to water stress, we performed differential expression analysis between the water-stress and control conditions’ RNA-seq data. Our results showed that 16,452 and 18,595 DEGs were identified in Colotana.PEG vs. Colotana.con (Supplementary Table S2) and Tincurrin.PEG vs. Tincurrin.con (Supplementary Table S3) comparisons, respectively. In Colotana.PEG vs. Colotana.con, 7173 genes were upregulated, while in Tincurrin.PEG vs. Tincurrin.con, 9109 genes were upregulated. Among the upregulated genes, 3692 and 5628 were specifically upregulated in Colotana.PEG vs. Colotana.con and Tincurrin.PEG vs. Tincurrin.con, respectively (Figure 1A). The number of downregulated genes was 9279 in Colotana.PEG vs. Colotana.con and 9486 in Tincurrin.PEG vs. Tincurrin.con. Among the downregulated genes, 4837 and 5044 were specifically downregulated in Colotana.PEG vs. Colotana.con and Tincurrin.PEG vs. Tincurrin.con, respectively (Figure 1B).

### 2.2. Validation of Gene Expression by qRT-PCR

**Figure 2:**
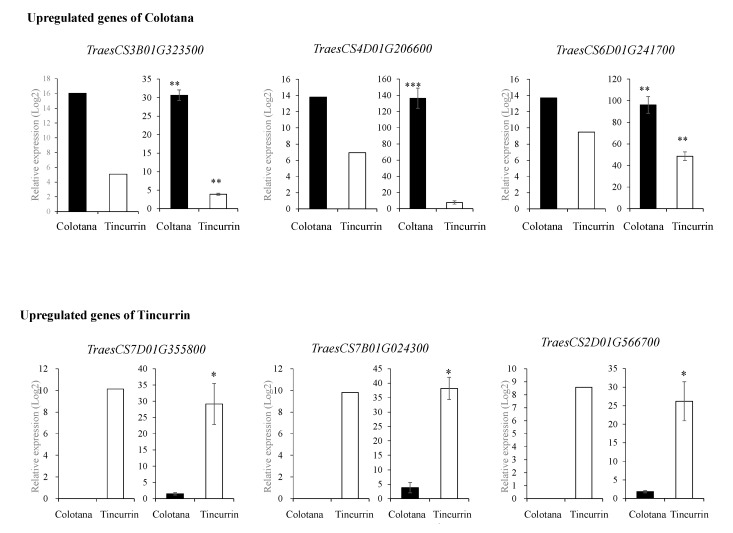
qRT-PCR validation of up- and downregulated DEGs in Colotana and Tincurrin. RNA-seq results (left) and qRT-PCR results (right) for six genes. Values are means ± SD of three biological replicates in qRT-PCR. The expression levels of each gene are expressed as relative to the mean value of the control samples. Student’s t-test was used to assess the significance of differences from control samples, * *p* < 0.05, ** *p* < 0.01, *** *p* < 0.001.

To validate the results of RNA-seq analysis, qRT-PCR was conducted on six randomly selected DEGs (*TraesCS3B01G323500*: *sulfate transporter*; *TraesCS4D01G206600*: *70 kDa heat shock protein*; *TraesCS6D01G241700*: *hydroxycinnamoyl-CoA shikimate/quinate hydroxycinnamoyltransferase*; *TraesCS7D01G355800*: *receptor-kinase*; *TraesCS7B01G024300*: *transmembrane protein*; *TraesCS2D01G566700*: *germin-like protein*) and compared with the results obtained from RNA-seq analysis (Figure 2 and Supplementary Table S4). The relative expression levels of these genes from both qRT-PCR and RNA-seq analysis were generally consistent, thus validating the RNA-seq data. Differences observed in fold changes measured by RNA-seq and qRT-PCR were consistent with previous studies [19].

### 2.3. Functional Gene Ontology (GO) Enrichment and Kyoto Encyclopedia of Genes and Genomes (KEGG) Pathway Analysis

To investigate the involvement of possible biological processes or pathways in water-stress response of the two genotypes, we performed gene ontology (GO) and Kyoto Encyclopedia of Genes and Genomes (KEGG) pathway enrichment analyses for specifically up- and downregulated genes in Colotana.PEG vs. Colotana.con and Tincurrin.PEG vs. Tincurrin.con comparisons. Among 3692 upregulated genes in Colotana, the top three highly enriched GO terms were generation of precursor metabolites and energy (GO:0006091), oxidative phosphorylation (GO:0006119), and oxidation reduction (GO:0055114), shown in Supplementary Table S5. The top three highly enriched GO categories in downregulated genes in Colotana were related to protein amino acid phosphorylation (GO:0006468), phosphorylation (GO:0016310), and phosphorus metabolic process (GO:0006793). The 5628 upregulated genes in Tincurrin showed overrepresentation of GO terms including protein amino acid phosphorylation (GO:0006468), phosphorylation (GO:0016310), and post-translational protein modification (GO:0043687). Cell wall organization or biogenesis (GO:0071554), response to oxidative stress (GO:0006979), and cellular polysaccharide metabolic process (GO:0044264) were the top three highly significant GO categories of downregulated genes in Tincurrin genotype.

KEGG pathway enrichment analysis showed that among upregulated genes in Colotana, the top three highly significant pathways (based on adjusted *p*-value) were involved in oxidative phosphorylation (118 genes), metabolic pathways (404 genes), and glutathione metabolism (51 genes) (Supplementary Table S6). Only two pathways, plant–pathogen interaction (57 genes) and plant hormone signal transduction (60 genes), were significant among downregulated genes in Colotana. For upregulated genes in Tincurrin, the highly enriched pathways were plant–pathogen interaction (67 genes), glutathione metabolism (37 genes), and plant hormone signal transduction (56 genes). Among downregulated genes in Tincurrin, the top three highly significant pathways were related to biosynthesis of secondary metabolites (263 genes), phenylpropanoid biosynthesis (71 genes), and metabolic pathways (368 genes). 

The enriched GO terms and KEGG pathways among specifically upregulated genes in Colotana (3692 genes) were mainly related to biosynthesis of metabolites and energy production, which are required for root growth; therefore, we focused on those genes to detect the corresponding candidate genes. Moreover, a BLASTP search for sequences with homology to wheat (*Triticum aestivum*), *Arabidopsis thaliana*, and rice (*Oryza sativa*) was performed, and only the top hit was selected in order to choose candidate genes related to root elongation and lateral root development under abiotic stress conditions. 

### 2.4. Upregulated Genes in Colotana Related to Root Growth under Abiotic Stress

Transcription factors (TFs) have regulatory roles in gene expression in response to physiological and environmental signals. The identified genes encoding TFs, including MYB (e.g., TraesCS1D01G275400), No apical meristem (NAM) protein (NAC, e.g., TraesCS3A01G406000), WRKY (e.g., TraesCS3B01G375200), basic helix-loop-helix (bHLH, e.g., TraesCS6B01G411300), and APETALA2/Ethylene-Responsive Factor (AP2, e.g., TraesCS2A01G288000), were upregulated only in Colotana under the water-stress condition (Supplementary Table S3). Late-embryogenesis-abundant (LEA) proteins are widely assumed to play critical roles in cellular dehydration tolerance [23]. Two DEGs encoded LEA proteins (e.g., TraesCS4D01G177500) among overexpressed genes in the Colotana genotype. Furthermore, pyrroline-5-carboxylate reductase (P5CR) is the rate-limiting enzyme in the proline (Pro) biosynthesis process [24], and one gene encoding P5CR (TraesCS3B01G538100) showed increased expression in the Colotana genotype. 

### 2.5. Identified Variants in Transcriptome of the Two Genotypes

We compared the transcriptome sequences of Colotana and Tincurrin with the wheat reference genome assembly (Chinese Spring) to investigate the polymorphic sites between the two genotypes. After additional filtration of low-ranked (mostly without impact on protein function) and modifier variants (usually non-coding), a total of 15,207 SNPs (moderate- and high-impact) and Indels (high impact) was detected between Colotana and Tincurrin genotypes by mapping to the Chinese Spring genome. The identified variants may influence the protein functions and resulting different root lengths between the two genotypes under water stress condition (Supplementary Table S7). The most frequent term was missense variant, which varied from 9942 in Colotana and 4520 in Tincurrin genotypes (Table 1 and Supplementary Table S7). Furthermore, among upregulated genes in Colotana, we detected one potential candidate gene *TraesCS2A01G102000* encoding NAC TF, harboring one missense variant, which may influence the drastic root length of Colotana under water stress condition. 

### 2.6. Bias of up- and Downregulated Homoeologous Genes in Response to Water Stress between Genotypes

**Figure 3:**
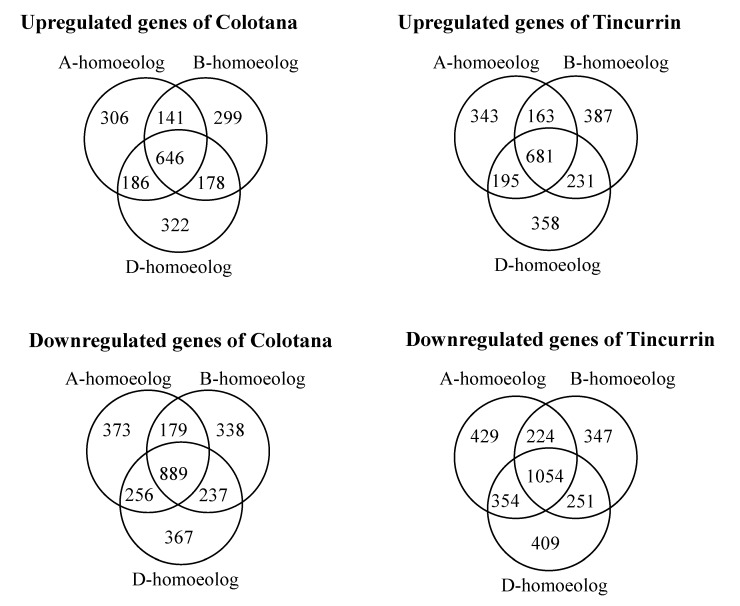
Classification of the differentially expressed genes in the A, B, and D subgenome after water stress in Colotana and Tincurrin genotypes. Venn diagram analysis showed relationship of the up- and downregulated homoeologous genes between the two genotypes.

Bread wheat is an allohexaploid containing three closely related subgenomes (A, B, and D), and which shows improved tolerance to abiotic stress relative to diploid and tetraploid wheat [25]. Although the underlying mechanisms are largely unknown, the coordinated expression of homoeologs in response to environmental stresses may play a critical role [1]. To unravel the different contribution of homoeologous genes in plants subjected to water stress, we compared the relative expression patterns of DEGs among 18,407 homoeologous gene loci that had exactly one representative member from each subgenome (referred to as homoeologous triads; 18,407 × 3 = 55,221 genes) in Colotana and Tincurrin under the water-stress condition and control condition, based on adjusted *p*-value (padj) < 0.05 and expression level (|log2 fold change| ≥ 2) criteria (Figure 3, Supplementary Table S8A,B). Among 18,407 triads, 4717 (25.6%) and 5426 (29.4%) exhibited differential expression of at least one homoeolog in response to water stress in Colotana and Tincurrin, respectively. Specifically, 306 (373) A-homoeologs, 299 (338) B-homoeologs, and 322 (367) D-homoeologs were upregulated (downregulated) in Colotana after a 6 h PEG treatment, whereas the corresponding numbers for Tincurrin were 343 (429) for A-homoeologs, 387 (347) for B-homoeologs, and 358 (409) for D-homoeologs, respectively. 

### 2.7. Various Expression Patterns of Homoeologous Genes under Water Stress in Two Genotypes

**Figure 4:**
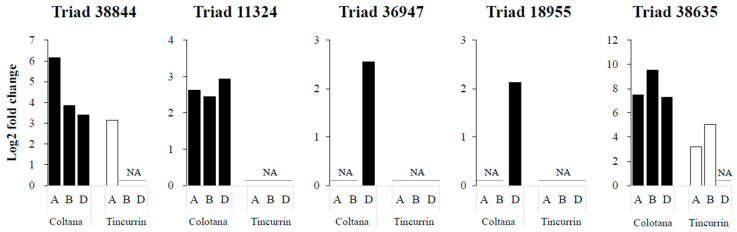
Difference of gene expression patterns of homoeologous genes under water stress in Colotana and Tincurrin genotypes. The relative expression of Triads 38,844, 11,324, 36,947, 18,955, and 38,635 in two genotypes is shown. The x-axis shows homoeologous genes and the y-axis shows relative expression. Each bar shows the relative expression in roots of Colotana (black) and Tincurrin (white) at 6 h.

TFs regulate signal transduction and gene expression patterns that probably function in the response to water stress. We identified various TFs (Supplementary Table S2) that may function in regulating some water-stress-responsive genes. Several TFs were characterized as upregulated under osmotic stress in other plants. Four TFs were selected as upregulated genes in Colotana (e.g., TraesCS6A01G057400: NAC TF; e.g., TraesCS7D01G540700: MYB TF; TraesCS7D01G497400: WRKY TF; TraesCS7D01G171300: bZIP domain TF) and expression of their homoeologs (Triads 38,844, 11,324, 36,947, and 18,955, respectively) were investigated between the two genotypes (Figure 4). An upregulated gene encoding detoxification enzyme (e.g., TraesCS3B01G471500: glutathione S-transferase) and the corresponding homoeolog (Triad 38,635) was also investigated between the two genotypes (Figure 4). The A-homoeolog of Triad 38,844, along with the A- and B-homoeologs of Triad 38,635, exhibited higher expression patterns in Colotana relative to those in Tincurrin. The expression levels of the three homoeologs of Triad 11,324 were all significantly upregulated in Colotana, but showed no expression in Tincurrin. Interestingly, Triad 11,324 is annotated as TaMYB32 in wheat, and its overexpression enhanced the salt tolerance in transgenic *A. thaliana* plants [26]. The D-homoeolog of Triads 36,947 and 18,955 were specifically upregulated in Colotana, but exhibited no expression in Tincurrin. Moreover, Triad 36,947, annotated as WRKY79 in wheat, enhances salinity tolerance in transgenic *A. thaliana* in an ABA-dependent pathway [27]. 

### 2.8. Divergent Expression of Homoeologous Genes in Response to Abiotic Stress

**Figure 5:**
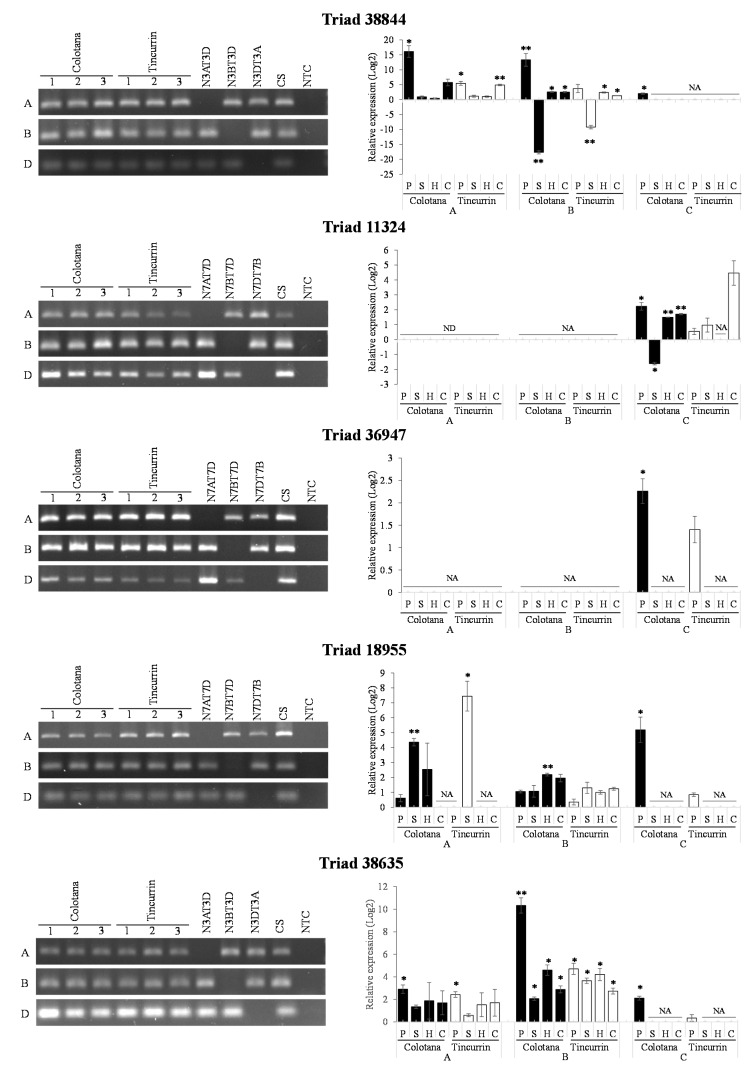
Comparison of differentially expressed A, B, and D homoeologous genes under abiotic stresses. Validation of expression divergence of homoeologous genes using qRT-PCR. PCR validation of qRT-PCR confirmed homoeologous genes using homoeolog-specific primers. The results of PCR experiments further confirmed the localization of homoeologous genes in their corresponding subgenomes. Student’s t-test was used to assess the significance of differences from control samples, * *p* < 0.05, ** *p* < 0.01. Each experiment was performed using Colotana and Tincurrin with three biological replicates. Chinese Spring (CS) was used as positive control, all reagents without template DNA as negative control (NTC). PEG-induced water stress: P; high-salinity stress: S; heat stress: H; cold stress: C; no data: ND; and no amplification: NA.

It is hypothesized that a gene that is responsive to certain stress may show differences in homoeologous gene expression in response to those conditions. To determine whether abiotic stress affects homoeologous gene expression, the homoeologs of five triads (Triads 38,844, 11,324, 36,947, 18,955, and 38,635) were examined under water, high-salinity, heat, and cold stresses through qRT-PCR analysis using homoeolog-specific primers that were validated via nullisomic–tetrasomic line detection (Figure 5). To ensure whether high-salinity, heat, and cold stresses were conducted properly, we used stress-specific index genes, which were reported previously (Supplementary Figure S2) [28,29,30]. Nullisomic–tetrasomic line detection indicated that our primers were homoeolog-specific and stress index genes showed that stress treatments were conducted properly. The expression patterns of homoeologs resulting from the qRT-PCR analysis under the water-stress condition was consistent with the observation made based on RNA-seq data (Figure 4 and Figure 5). Interestingly, homoeologous genes showed stress-specific patterns. For example, the D-homoeolog of Triad 36,947 was significantly induced by the water-stress condition in the Colotana genotype, while the A and B homoeologs exhibited no expression in all samples. The expression levels of the A and D homoeologs of Triad 38,635 were low in all stress treatments in both genotypes, but the B homoeolog was significantly upregulated under all stress treatments in both genotypes. Interestingly, the B homoeolog of Triad 38,844 was downregulated by high-salinity stress while it was upregulated by heat and cold stresses in both genotypes. Similarly, the expression of the D homoeolog of Triad 11,324 was downregulated by high-salinity stress, while its abundance was induced by water, heat, and cold stresses in the Colotana genotype. We could not acquire the gene expression data for the B homoeolog of Triad 11,324 (*TraesCS7B01G478200*), even though we used five primer sets. The reason is considered to the low expression level of the corresponding gene in the Colotana genotype. The A homoeolog of Triad 18,955 exhibited upregulation after high-salinity stress in both genotypes, while its B and D homoeologs were specifically upregulated in the Colotana genotype by heat and water stresses, respectively.

## 3. Discussion

Roots play a critical role in water and mineral acquisition, as well as being the first organs that perceive moisture deficit in the soil. Previously, it was reported that drought-tolerant genotypes had longer roots and more lateral roots in order to absorb soil water efficiently [17,31]. Our previous studies showed that Colotana had longer roots than the susceptible Tincurrin under water-stress conditions [20,21], which could assist plants with the Colotana genotype to take up more water from a drying soil’s deeper layers. Transcriptomic approaches can contribute to deciphering differential gene expression between contrasting varieties/genotypes and be used to detect genetic variants such as SNPs and Indels. In recent years, comparative transcriptome studies have been used to discover candidate genes for various types of abiotic stress tolerance in rice [32] and barley [33]. In order to further explore the mechanisms of wheat tolerance against water deficits at the molecular level, the early transcriptomic response of drought-tolerant Colotana and drought-susceptible Tincurrin genotypes were evaluated. Clearly, there were distinct differences in the transcriptome levels between the two genotypes in their responses to water stress. 

### 3.1. Candidate Genes Involved in Root Development under Abiotic Stress

TFs regulate gene expression in response to environmental and physiological signals [4]. Germination and root length assays of transgenic *A. thaliana* seedlings overexpressing wheat TaNAC29 TF exhibited enhanced tolerances to high-salinity and dehydration stresses. Moreover, it was demonstrated that TaNAC29 participated in the ABA-mediated pathway, and activated antioxidant enzymes to improve plant tolerance [34]. The ectopic expression of TaWRKY1 and TaWRKY33 activated several stress-related downstream genes, increased germination rates, and promoted root growth in *A. thaliana* under various abiotic stresses [35]. The salt-inducible TaMYB32, TaMYB73, and TaWRKY79 enhanced root length and salt-stress tolerance in *A. thaliana* seedlings [26,27,36]. Rice seedlings overexpressing OsNAC6 TF showed increased root number, root diameter with large aerenchymal cells and enhanced drought tolerance [37]. Studies have shown that TabHLH39 can improve the stress resistance of *A. thaliana* transgenic plants by increasing the content of soluble sugar and Pro and lowering the level of electrolyte leakage compared with wild-type plants under drought and salt-stress treatments [38]. Discovering stress-responsive genes is crucial for breeding stress-tolerant wheat plants through transgenic approaches. In this work, water-stress-induced TFs were identified in wheat, and their homologous gene functions under abiotic stresses in *A. thaliana* and rice (*O. sativa*) were reported. Based on the performance of transgenic plants, it was proposed that overexpression of TFs led to improved abiotic-stress tolerance through an integrated effect of the regulation of stress-responsive genes and changes in some physiological traits that were triggered. Therefore, we can hypothesize that the upregulated TFs in Colotana may contribute to the enhanced root length and water-stress tolerance of this genotype. 

Higher plants accumulate free Pro under osmotic stress, and P5CR is the last enzyme in the cascade for Pro synthesis [39]. In our study, we identified the *P5CR* gene among the overrepresented GO terms, and found KEGG pathways such as proline metabolic process (GO:0006560), proline biosynthetic process (GO:0006561), biosynthesis of secondary metabolites, and biosynthesis of amino acids pathways in upregulated genes in the Colotana genotype (Supplementary Tables S5 and S6). Studies showed that transgenic *A. thaliana* plants overexpressing the *TaP5CR* gene can enhance root growth under salt stress, increase Pro content, and decrease MDA content under NaCl, PEG, and ABA stress treatments [39]. The higher expression pattern of *P5CR* is reported to be consistent with the function of Pro as an energy, nitrogen, and carbon source and as an osmoticum in response to dehydration stress [40]. Decreased MDA content suggested that the product of the exogenous *TaP5CR* gene has a role in a protective antioxidation system and functions to reduce oxidative damage to the transgenic *A. thaliana* plants. Therefore, we can hypothesize that *P5CR* gene may play a crucial role in enhancing root growth and water-stress tolerance, as well as reducing oxidative damage in the Colotana genotype. 

LEA proteins contribute in tolerance to drought, high-salinity, and cold stresses in different organisms. Transgenic rice seedlings overexpressing the *OsLEA3-2* gene showed stronger root growth performance under water stress relative to the control [41]. It has been reported that LEA proteins are associated with protective functions, specifically in dehydrated tissues, where they are considered to act as chaperones, protecting other proteins from aggregation or desiccation [42]. Therefore, we can hypothesize that this group of proteins may underlie an adaptation strategy to osmotic stress in Colotana roots. 

These results enhance our understanding of transcriptomic response of wheat roots to water-stress conditions and provide candidate genes for wheat breeding programs. 

### 3.2. Effects of SNPs on Water Stress Response 

SNPs are the most abundant form of polymorphism in DNA sequences and are useful in genetic studies [43]. Application of transcriptome sequencing has become a cost-effective method for the detection of SNPs with high accuracy. Functional genes can be sequenced at high coverage and the SNPs in coding genes can be detected [44,45]. A nonsynonymous or missense variant can cause an amino acid change in a protein and a drastic phenotypic effect [46]. We identified one DEG encoding NAC TF with a missense variant in the Colotana genotype (Supplementary Table S7). It is also possible that the SNP detected in NAC TF in our study may affect the drastic root length growth under water stress between the two genotypes. Furthermore, the detected SNPs and Indels in the transcriptome profile between the two genotypes could enable us to develop molecular markers for crop breeding studies.

### 3.3. Association of Differential Expression of Homoeologous Genes with Abiotic Stress Acclimation

Polyploidy often leads to changes in gene expression [47]. A fraction of expressed homoeologs in allopolyploids are likely to respond differently when subjected to stresses. For example, in allotetraploid cotton (*G. hirsutum*), it was reported that 23 out of 30 examined genes exhibited variation in the contribution of homoeologous genes to heat, cold, drought, high-salinity, and water-submersion stresses, possibly due to epigenetic modification or regulatory region variation [22]. Consistently, in our study, five examined triads showed variation in the contribution of homoeologous genes to water, high-salinity, heat, and cold stresses in the two genotypes. Hexaploid bread wheat has been reported to show improved tolerance to stress conditions compared to its tetraploid wheat progenitor (*Triticum turgidum* L.) [48]. Therefore, we can hypothesize that expression divergence of the three homoeologous genes might enable wheat plants to better cope with various stresses in the natural environment.

In summary, the present study reported transcriptional regulation of water-stress-induced root growth in wheat at the seedling stage. Colotana roots showed better adaptation to water stress by upregulation of genes for TFs, i.e., NAC, WRKY, MYB, bHLH, P5CR, and protective protein LEA, which are important for enhanced water-stress tolerance and root growth under stress. We also identified various SNPs and Indels between the transcriptome profiles of two genotypes, which may affect their drastic differences root growth under water stress. The detected DEGs and genes harboring variants could enable us to develop markers for crop breeding studies. Finally, the expression pattern of homoeologous genes under various abiotic stresses were evaluated in the two genotypes. The variation of homoeologous gene expression in response to environmental stresses may enable plants to better cope with stresses in their natural environments.

## 4. Materials and Methods

### 4.1. Plant Growth and Treatment

The two test genotypes in this study, Colotana (AGG20118WHEA1) and Tincurrin (AGG20578WHEA1), were previously identified as water-stress-tolerant and -susceptible, respectively [20,21]. Seeds of these genotypes were germinated and grown in a customized hydroponic system following Ayalew et al. [20]. In addition to water, high-salinity, heat, and cold stresses tolerance tests were separately conducted in parallel to test homoeologous variations in gene expression levels between genotypes. Seven day old seedlings were subjected to water (−0.82 MPa), high-salinity (200 mM NaCl), heat (40 °C), and cold stresses (4 °C) for 6 h and 72 h, separately. Each treatment set-up was supplied with half-strength Hoagland’s solution. The control condition (con) was grown in half-strength Hoagland’s solution. The pH of the solution was adjusted to 5.5–5.7 with relative humidity 65–70% and day/night temperature 25/22 °C under 14/10 h day/night photoperiod. Fresh root samples were collected after 6 h and 72 h of stress treatments and immediately frozen in the liquid nitrogen and stored at −80 °C for further use.

### 4.2. RNA Isolation, Library Preparation, and RNA Sequencing

Total RNA was extracted from root tissues (12 in total, two genotypes (Colotana and Tincurrin) × two treatments (PEG-induced water stress and control) × three biological replicates) using the RNeasy Plant Mini Kit (Qiagen, Hilden, Germany) according to the manufacturer’s instructions. Construction of 12 cDNA libraries (two genotypes, two treatments, and three replicates) from total RNA using a TruSeq RNA sample preparation kit (Illumina Inc., San Diego, CA, USA) was performed according to the manufacturer’s protocol and they were sequenced with an Illumina platform in Novogene Bioinformatics Technology Co. Ltd., Beijing, China to generate 150 bp paired-end reads.

### 4.3. Quality Control, Mapping of RNA-Seq Reads, and Variant Calling

The quality of raw sequencing data was assessed using FastQC v0.10.1 (http://www.bioinformatics.babraham.ac.uk/projects/fastqc/). Clean reads were obtained by removing low-quality bases from both the 5′ and 3′ ends of paired-end reads with the settings minimum read length > 70, sliding window = 25 bp, and average quality > 25 in Trimmomatic v0.32 [49]. The wheat genome assembly (Chinese Spring RefSeqv1.0) was downloaded from the International Wheat Genome Sequencing Consortium (IWGSC) [50]. The construction of an index for the reference genome was performed using Bowtie2 v2.3.4.1, and paired-end clean reads were aligned to the reference genome using TopHat v2.1.1 followed by sorting the alignments by SAMTools v1.8 [51,52]. Due to contrasting difference between the two genotypes and to increase the alignment rate, the number of allowed mismatches were determined to be three, using the command “-N 3” in TopHat v2.1.1. The multi-hits were prohibited in our analysis using the command “-g 1”. The obtained reads were deposited in the National Center for Biotechnology Information (NCBI) under the BioProject ID PRJNA594809 with the Sequence Read Archive (SRA) submission ID SUB6661743. SAMTools mpileup function, and BCFTools variant caller, with a minimum Phred quality of 20 and a maximum read depth of 100 default parameters, were used to filter the variants [51]. The resulting VCF file was filtered by removing heterozygous variants using a custom perl-script. SnpEff software was used to identify changes in amino acid function resulting from nucleotide substitutions [53,54].

### 4.4. Detection of DEGs, and GO and KEGG Pathway Enrichment Analysis

The number of reads mapped to each gene was counted using HTSeq-counts v0.9.1 [55]. The DESeq2 R package v3.7 was used to estimate DEG analysis, and genes with an adjusted *p*-value (padj) < 0.05 and expression level (|log2 fold change| ≥ 2) were considered DEGs for further analyses [56].

We selected the best hits based on a BLASTP search against NCBI Nr protein database and an E-value 1E-08 cut-off, and, by using IWGSC functional annotation (iwgsc refseqv1.0 Functional Annotation v1 HC genes v1.0) file, we assigned description, GO, and Pfam IDs for each gene. GO enrichment analysis for up- and downregulated DEGs in the biological process category was implemented using the BiNGO tool with hypergeometric test and Benjamini and Hochberg false discovery rate (FDR) to obtain an adjusted *p*-value (<0.05) cut-off [57]. The KOBAS software was used to perform KEGG pathway enrichment analysis according to *O. sativa* database with adjusted *p*-value < 0.05 cut-off [58].

### 4.5. Validation of RNA-Seq Data by qRT-PCR and Chromosome Location of Homoeologous Genes

The first-strand cDNA was synthesized using the Transcriptor First Strand cDNA synthesis kit (Roche, Basel, Switzerland) according to the manufacturer’s instructions. The CFX96 Real-Time system (Bio-Rad, USA) and THUNDERBIRD SYBR qPCR Mix kit (TOYOBO, Japan) were used for qRT-PCR, with primer sets shown in Supplementary Table S4. The wheat Actin gene was used as an internal reference.

To evaluate the expression patterns of homoeologous genes, we used the high-confidence (HC) wheat homoeologous gene set prepared by Ramírez-González et al. [59] (Supplementary Table S8A,B). The nullisomic–tetrasomic lines were used to confirm the homoeolog-specificity of the designed primers [60], using PCR amplification and 2% agarose gel stained with ethidium bromide.

## Figures and Tables

**Table 1:** Summary of variant calling between Colotana and Tincurrin.

Variant Category	Effect (Sequence Ontology)	Impact	Colotana	Tincurrin	All
SNPs	Missense_variant	Moderate	9942	4520	14,462
	Missense_variant and splice_region_variant	Moderate	177	65	242
	Stop_gained	High	96	71	167
	Splice_acceptor_variant and intron_variant	High	25	35	60
	Splice_donor_variant and intron_variant	High	11	48	59
	Stop_lost and splice_region_variant	High	18	17	35
	Start_lost	High	6	6	12
	Stop_lost	High	3	4	7
	Splice_acceptor_variant and splice_donor_variant and intron_variant	High	3	0	3
Indels	Frameshift_variant	High	95	33	128
	Frameshift_variant and splice_region_variant	High	6	16	22
	Stop_gained and splice_region_variant	High	1	3	4
	Frameshift_variant and stop_lost and splice_region_variant	High	1	1	2
	Frameshift_variant and splice_donor_variant and splice_region_variant and intron_variant	High	0	2	2
	Frameshift_variant and start_lost	High	1	0	1
	Frameshift_variant and stop_gained and splice_region_variant	High	1	0	1
	Total variant sites	-	10,386	4821	15,207

## Supplementary Materials

The following are available online at https://www.mdpi.com/2223-7747/9/5/596/s1, Figure S1A: Pearson’s correlation coefficients and PCA plot analyses among biological replicates, Figure S1B: The expression profile of five triads detected via RNA-seq technique under water stress in Colotana.PEG vs. Colotana.con and Tincurrin.PEG vs. Tincurrin.con comparisons, Figure S2: qRT-PCR analysis of three index genes for high-salinity, heat, and cold stresses after a 6 h treatment in Colotana and Tincurrin, Table S1: Mapping of RNA-seq reads obtained from root samples into the reference Chinese_Spring_v1.0_pseudomolecules_parts. fasta genome sequence, Table S2: List of DEGs between Colotana.PEG and Colotana.con, Table S3: List of DEGs between Tincurrin.PEG and Tincurrin.con, Table S4: Designed PCR primers for qRT-PCR analysis, Table S5: Significantly enriched GO terms for up- and downregulated DEGs in Colotana and Tincurrin, Table S6: Significantly enriched KEGG pathways for up- and downregulated DEGs in Colotana and Tincurrin, Table S7: Complete list of identified variants between Colotana and Tincurrin, Table S8A: Homoeologous genes up/ down regulated between Colotana.PEG vs. Colotana.con., Table S8B: Homoeologous genes up/down regulated between Tincurrin.PEG vs. Tincurrin.con.

## References

[1] Liu Z., Xin M., Qin J., Peng H., Ni Z., Yao Y., Sun Q. (2015). Temporal transcriptome profiling reveals expression partitioning of homeologous genes contributing to heat and drought acclimation in wheat (*Triticum aestivum* L.). BMC Plant Biol..

[2] Gill B.S., Appels R., Botha-Oberholster A.-M., Buell C.R., Bennetzen J.L., Chalhoub B., Chumley F., Dvorák J., Iwanaga M., Keller B. (2004). A workshop report on wheat genome sequencing: International Genome Research on Wheat Consortium. Genetics.

[3] Brisson N., Gate P., Gouache D., Charmet G., Oury F.-X., Huard F. (2010). Why are wheat yields stagnating in Europe? A comprehensive data analysis for France. Field Crops Res..

[4] Budak H., Kantar M., Yucebilgili Kurtoglu K. (2013). Drought Tolerance in Modern and Wild Wheat. Sci. World J..

[5] Chloupek O., Dostál V., Středa T., Psota V., Dvořáčková O. (2010). Drought tolerance of barley varieties in relation to their root system size. Plant Breed..

[6] Lilley J.M., Kirkegaard J.A. (2011). Benefits of increased soil exploration by wheat roots. Field Crops Res..

[7] Palta J., Chen X., Milroy S., Rebetzke G., Dreccer M., Watt M. (2011). Large root systems: Are they useful in adapting wheat to dry environments?. Funct. Plant Biol..

[8] Sengupta D., Kannan M., Reddy A.R. (2011). A root proteomics-based insight reveals dynamic regulation of root proteins under progressive drought stress and recovery in *Vigna radiata* (L.) Wilczek. Planta.

[9] Rabello A.R., Guimarães C.M., Rangel P.H.N., da Silva F.R., Seixas D., de Souza E., Brasileiro A.C.M., Spehar C.R., Ferreira M.E., Mehta Â. (2008). Identification of drought-responsive genes in roots of upland rice (*Oryza sativa* L.). BMC Genomics.

[10] Gao Y., Lynch J.P. (2016). Reduced crown root number improves water acquisition under water deficit stress in maize (*Zea mays* L.). J. Exp. Bot..

[11] Venuprasad R., Shashidhar H.E., Hittalmani S., Hemamalini G.S. (2002). Tagging quantitative trait loci associated with grain yield and root morphological traits in rice (*Oryza sativa* L.) under contrasting moisture regimes. Euphytica.

[12] Uga Y., Sugimoto K., Ogawa S., Rane J., Ishitani M., Hara N., Kitomi Y., Inukai Y., Ono K., Kanno N. (2013). Control of root system architecture by DEEPER ROOTING 1 increases rice yield under drought conditions. Nat Genet..

[13] Ni Z., Kim E.-D., Ha M., Lackey E., Liu J., Zhang Y., Sun Q., Chen Z.J. (2009). Altered circadian rhythms regulate growth vigour in hybrids and allopolyploids. Nature.

[14] Comai L. (2005). The advantages and disadvantages of being polyploid. Nat. Rev. Genet..

[15] Chen Z.J. (2007). Genetic and epigenetic mechanisms for gene expression and phenotypic variation in plant polyploids. Annu. Rev. Plant Biol..

[16] Dalal M., Sahu S., Tiwari S., Rao A.R., Gaikwad K. (2018). Transcriptome analysis reveals interplay between hormones, ROS metabolism and cell wall biosynthesis for drought-induced root growth in wheat. Plant Physiol. Bioch..

[17] Hu L., Xie Y., Fan S., Wang Z., Wang F., Zhang B., Li H., Song J., Kong L. (2018). Comparative analysis of root transcriptome profiles between drought-tolerant and susceptible wheat genotypes in response to water stress. Plant Sci..

[18] Iquebal M.A., Sharma P., Jasrotia R.S., Jaiswal S., Kaur A., Saroha M., Angadi U.B., Sheoran S., Singh R., Singh G.P. (2019). RNAseq analysis reveals drought-responsive molecular pathways with candidate genes and putative molecular markers in root tissue of wheat. Sci. Rep..

[19] Chaichi M., Sanjarian F., Razavi K., Gonzalez-Hernandez J.L. (2019). Analysis of transcriptional responses in root tissue of bread wheat landrace (*Triticum aestivum* L.) reveals drought avoidance mechanisms under water scarcity. PLoS ONE.

[20] Ayalew H., Ma X., Yan G. (2015). Screening Wheat (*Triticum* spp.) Genotypes for Root Length under Contrasting Water Regimes: Potential Sources of Variability for Drought Resistance Breeding. J. Agron. Crop. Sci..

[21] Ayalew H., Liu H., Yan G. (2016). Quantitative analysis of gene actions controlling root length under water stress in spring wheat (*Triticum aestivum* L.) genotypes. Crop Pasture Sci..

[22] Dong S., Adams K.L. (2011). Differential contributions to the transcriptome of duplicated genes in response to abiotic stresses in natural and synthetic polyploids. New Phytol..

[23] Hundertmark M., Hincha D.K. (2008). LEA (late embryogenesis abundant) proteins and their encoding genes in *Arabidopsis thaliana*. BMC Genomics.

[24] Szoke A., Miao G.H., Hong Z., Verma D.P. (1992). Subcellular location of delta-pyrroline-5-carboxylate reductase in root/nodule and leaf of soybean. Plant Physiol..

[25] Dubcovsky J., Dvorak J. (2007). Genome Plasticity a Key Factor in the Success of Polyploid Wheat Under Domestication. Science.

[26] Zhang L., Zhao G., Jia J., Liu X., Kong X. (2012). Molecular characterization of 60 isolated wheat MYB genes and analysis of their expression during abiotic stress. J. Exp. Bot..

[27] Qin Y., Tian Y., Han L., Yang X. (2013). Constitutive expression of a salinity-induced wheat WRKY transcription factor enhances salinity and ionic stress tolerance in transgenic *Arabidopsis thaliana*. Biochem. Bioph. Res. Co..

[28] Zhang J.-F., Xu Y.-Q., Dong J.-M., Peng L.-N., Feng X., Wang X., Li F., Miao Y., Yao S.-K., Zhao Q.-Q. (2018). Genome-wide identification of wheat (*Triticum aestivum*) expansins and expansin expression analysis in cold-tolerant and cold-sensitive wheat cultivars. PLoS ONE.

[29] Xue G.-P., Drenth J., McIntyre C.L. (2015). TaHsfA6f is a transcriptional activator that regulates a suite of heat stress protection genes in wheat (*Triticum aestivum* L.) including previously unknown Hsf targets. J. Exp. Bot..

[30] Rahaie M., Xue G.P., Naghavi M.R., Alizadeh H., Schenk P.M. (2010). A MYB gene from wheat (*Triticum aestivum* L.) is up-regulated during salt and drought stresses and differentially regulated between salt-tolerant and sensitive genotypes. Plant Cell Rep..

[31] Moumeni A., Satoh K., Kondoh H., Asano T., Hosaka A., Venuprasad R., Serraj R., Kumar A., Leung H., Kikuchi S. (2011). Comparative analysis of root transcriptome profiles of two pairs of drought-tolerant and susceptible rice near-isogenic lines under different drought stress. BMC Plant Biol..

[32] Degenkolbe T., Do P.T., Zuther E., Repsilber D., Walther D., Hincha D.K., Köhl K.I. (2009). Expression profiling of rice cultivars differing in their tolerance to long-term drought stress. Plant Mol. Biol..

[33] Guo P., Baum M., Grando S., Ceccarelli S., Bai G., Li R., von Korff M., Varshney R.K., Graner A., Valkoun J. (2009). Differentially expressed genes between drought-tolerant and drought-sensitive barley genotypes in response to drought stress during the reproductive stage. J. Exp. Bot..

[34] Huang Q., Wang Y., Li B., Chang J., Chen M., Li K., Yang G., He G. (2015). TaNAC29, a NAC transcription factor from wheat, enhances salt and drought tolerance in transgenic *Arabidopsis*. BMC Plant Biol..

[35] He G.H., Xu J.Y., Wang Y.X., Liu J.M., Li P.S., Chen M., Ma Y.Z., Xu Z.S. (2016). Drought-responsive WRKY transcription factor genes TaWRKY1 and TaWRKY33 from wheat confer drought and/or heat resistance in *Arabidopsis*. BMC Plant Biol..

[36] He Y., Li W., Lv J., Jia Y., Wang M., Xia G. (2012). Ectopic expression of a wheat MYB transcription factor gene, TaMYB73, improves salinity stress tolerance in *Arabidopsis thaliana*. J. Exp. Bot..

[37] Lee D.K., Chung P.J., Jeong J.S., Jang G., Bang S.W., Jung H., Kim Y.S., Ha S.H., Choi Y.D., Kim J.K. (2017). The rice OsNAC6 transcription factor orchestrates multiple molecular mechanisms involving root structural adaptions and nicotianamine biosynthesis for drought tolerance. Plant Biotechnol. J..

[38] Zhai Y., Zhang L., Xia C., Fu S., Zhao G., Jia J., Kong X. (2016). The wheat transcription factor, TabHLH39, improves tolerance to multiple abiotic stressors in transgenic plants. Biochem. Bioph. Res. Co..

[39] Ma L., Zhou E., Gao L., Mao X., Zhou R., Jia J. (2008). Isolation, expression analysis and chromosomal location of P5CR gene in common wheat (*Triticum aestivum* L.). S. Afr. J. Bot..

[40] Hua X.J., van de Cotte B., Van Montagu M., Verbruggen N. (1997). Developmental Regulation of Pyrroline-5-Carboxylate Reductase Gene Expression in *Arabidopsis*. Plant Physiol..

[41] Duan J., Cai W. (2012). OsLEA3-2, an abiotic stress induced gene of rice plays a key role in salt and drought tolerance. PLoS ONE.

[42] Tunnacliffe A., Wise M.J. (2007). The continuing conundrum of the LEA proteins. Naturwissenschaften.

[43] Hinds D.A., Stuve L.L., Nilsen G.B., Halperin E., Eskin E., Ballinger D.G., Frazer K.A., Cox D.R. (2005). Whole-Genome Patterns of Common DNA Variation in Three Human Populations. Science.

[44] Helyar S.J., Limborg M.T., Bekkevold D., Babbucci M., van Houdt J., Maes G.E., Bargelloni L., Nielsen R.O., Taylor M.I., Ogden R. (2012). SNP Discovery Using Next Generation Transcriptomic Sequencing in Atlantic Herring (*Clupea harengus*). PLoS ONE.

[45] Yu Y., Wei J., Zhang X., Liu J., Liu C., Li F., Xiang J. (2014). SNP Discovery in the Transcriptome of White Pacific Shrimp Litopenaeus vannamei by Next Generation Sequencing. PLoS ONE.

[46] Ng P.C., Henikoff S. (2006). Predicting the Effects of Amino Acid Substitutions on Protein Function. Annu. Rev. Genomics Hum. Genet..

[47] Adams K.L., Wendel J.F. (2005). Polyploidy and genome evolution in plants. Curr. Opin. Plant Biol..

[48] Yang C., Zhao L., Zhang H., Yang Z., Wang H., Wen S., Zhang C., Rustgi S., von Wettstein D., Liu B. (2014). Evolution of physiological responses to salt stress in hexaploid wheat. Proc. Natl. Acad. Sci. USA.

[49] Bolger A.M., Lohse M., Usadel B. (2014). Trimmomatic: A flexible trimmer for Illumina sequence data. Bioinformatics.

[50] Alaux M., Rogers J., Letellier T., Flores R., Alfama F., Pommier C., Mohellibi N., Durand S., Kimmel E., Michotey C. (2018). Linking the International Wheat Genome Sequencing Consortium bread wheat reference genome sequence to wheat genetic and phenomic data. Genome Biol..

[51] Li H., Handsaker B., Wysoker A., Fennell T., Ruan J., Homer N., Marth G., Abecasis G., Durbin R., 1000 Genome Project Data Processing Subgroup (2009). The Sequence Alignment/Map format and SAMtools. Bioinformatics.

[52] Kim D., Pertea G., Trapnell C., Pimentel H., Kelley R., Salzberg S.L. (2013). TopHat2: Accurate alignment of transcriptomes in the presence of insertions, deletions and gene fusions. Genome Biol..

[53] Langmead B., Salzberg S.L. (2012). Fast gapped-read alignment with Bowtie 2. Nat. Methods.

[54] Cingolani P., Platts A., Wang L.L., Coon M., Nguyen T., Wang L., Land S.J., Lu X., Ruden D.M. (2012). A program for annotating and predicting the effects of single nucleotide polymorphisms, SnpEff: SNPs in the genome of Drosophila melanogaster strain w1118; iso-2; iso-3. Fly.

[55] Anders S., Pyl P.T., Huber W. (2014). HTSeq—a Python framework to work with high-throughput sequencing data. Bioinformatics.

[56] Love M.I., Huber W., Anders S. (2014). Moderated estimation of fold change and dispersion for RNA-seq data with DESeq2. Genome Biol..

[57] Maere S., Heymans K., Kuiper M. (2005). BiNGO: A Cytoscape plugin to assess overrepresentation of Gene Ontology categories in Biological Networks. Bioinformatics.

[58] Xie C., Mao X., Huang J., Ding Y., Wu J., Dong S., Kong L., Gao G., Li C.-Y., Wei L. (2011). KOBAS 2.0: A web server for annotation and identification of enriched pathways and diseases. Nucleic Acids Res.

[59] Ramírez-González R.H., Borrill P., Lang D., Harrington S.A., Brinton J., Venturini L., Davey M., Jacobs J., van Ex F., Pasha A. (2018). The transcriptional landscape of polyploid wheat. Science.

[60] Sears E. (1966). Nullisomic-tetrasomic combinations in hexaploid wheat. Chromosome Manipulations and Plant Genetics.

