# Supplementary figures and images for: Comparative Analysis of Root Transcriptome Reveals Candidate Genes and Expression Divergence of Homoeologous Genes in Response to Water Stress in Wheat
Source: Plants (Basel). 2020 May 7;9(5):596. doi: 10.3390/plants9050596 (PMC7284651; doi:10.3390/plants9050596)

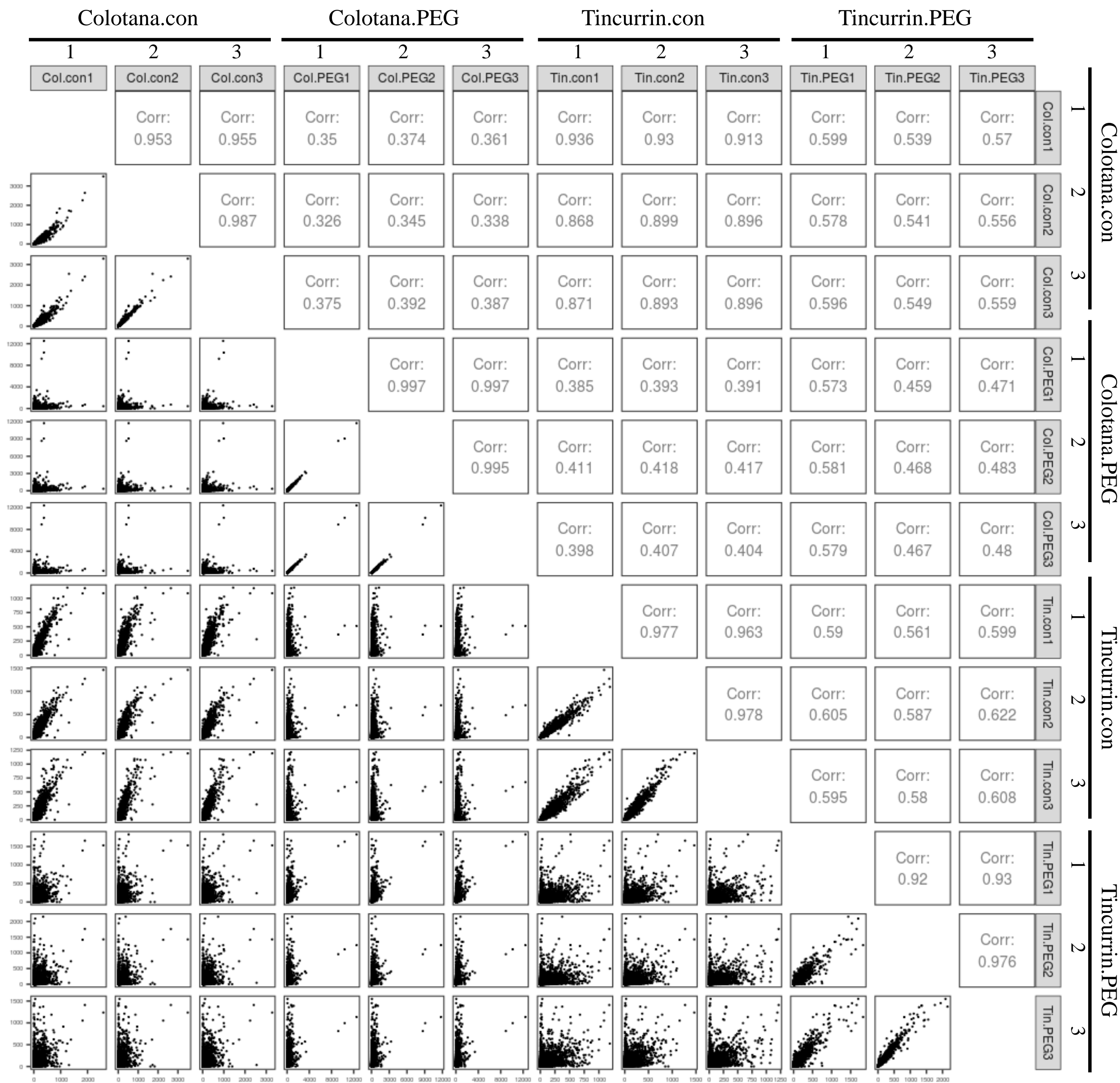

Figure S1A

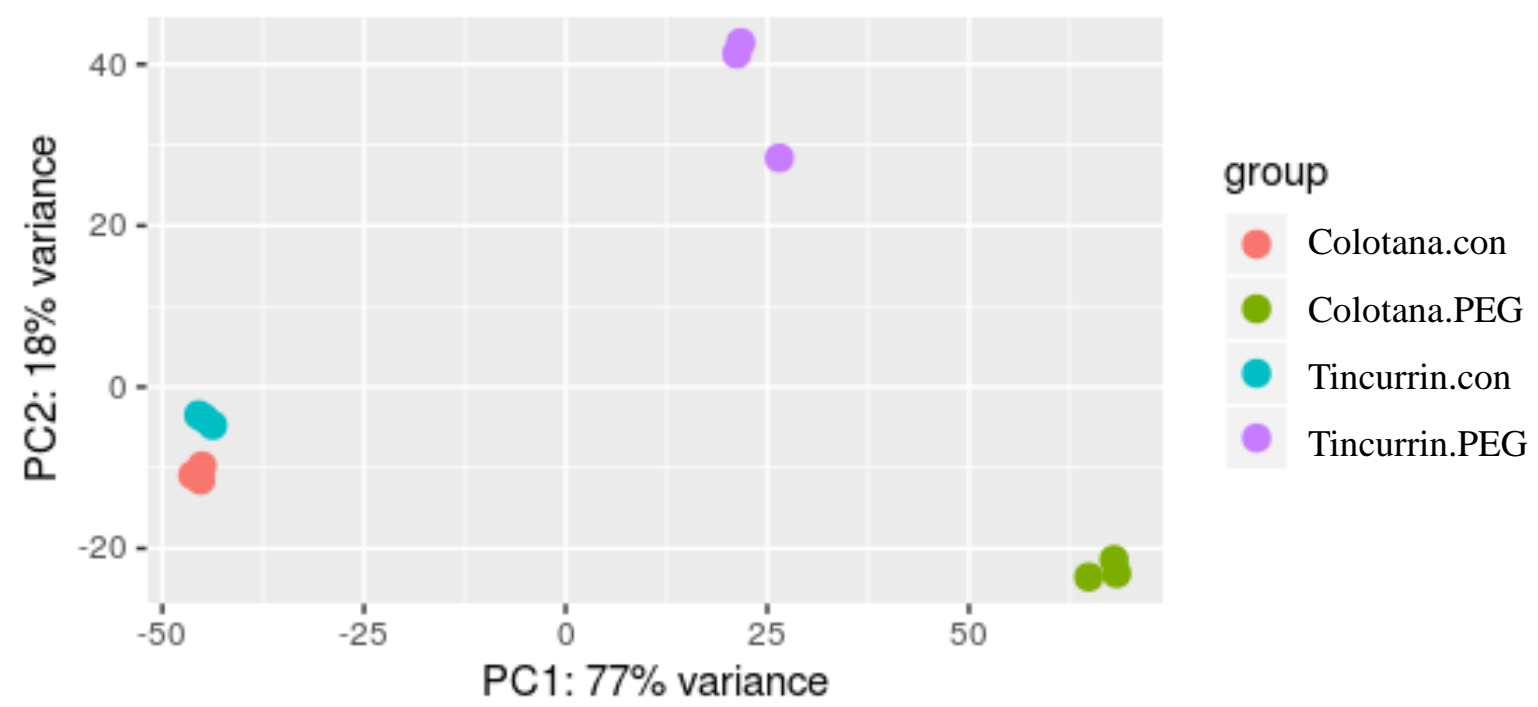

**Figure S1B**

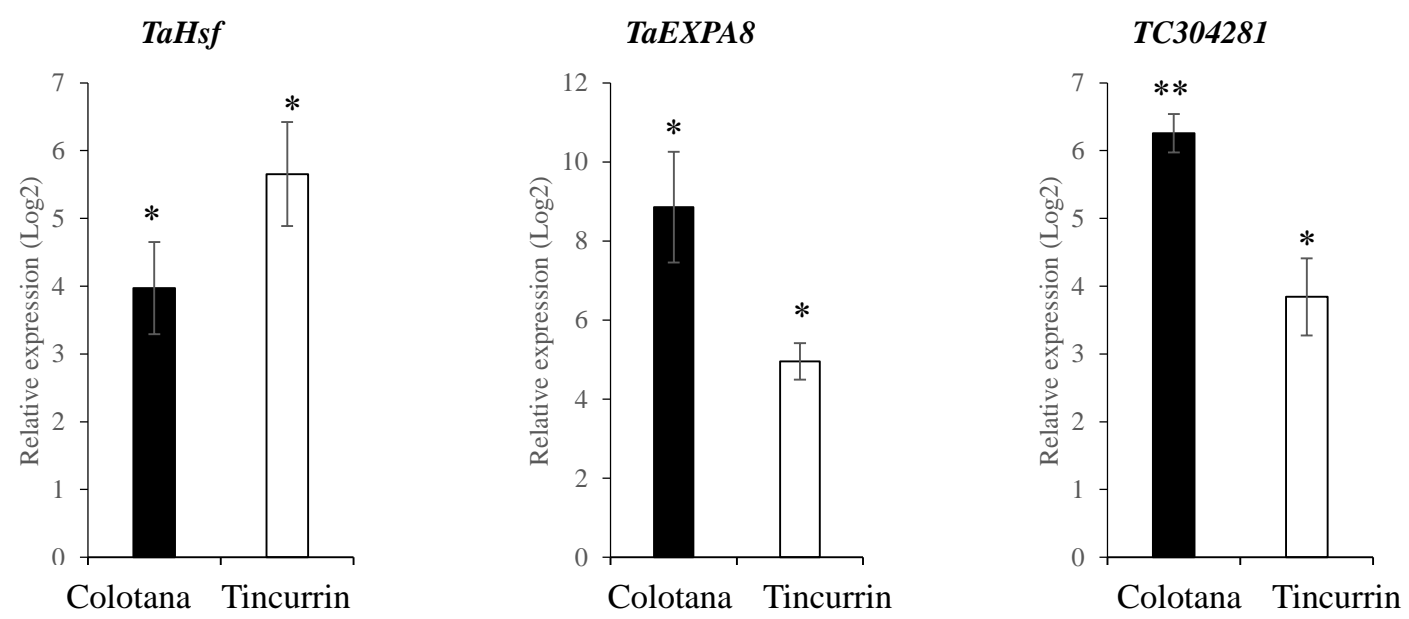

**Figure S2**

Supplement: Supplementary file 1 [file plants-09-00596-s001.zip › Supplementary files_Behnam et al/Supp.Figures.pdf]
